# MUC13 drives cancer aggressiveness and metastasis through the YAP1-dependent pathway

**DOI:** 10.26508/lsa.202301975

**Published:** 2023-10-04

**Authors:** Kyle Doxtater, Manish K Tripathi, Radhika Sekhri, Bilal B Hafeez, Sheema Khan, Nadeem Zafar, Stephen W Behrman, Murali M Yallapu, Meena Jaggi, Subhash C Chauhan

**Affiliations:** 1 Department of Immunology and Microbiology, School of Medicine, University of Texas Rio Grande Valley , McAllen, TX, USA; 2 South Texas Center of Excellence in Cancer Research, School of Medicine, University of Texas Rio Grande Valley , McAllen, TX, USA; 3 Department of Pathology, Montefiore Medical Center College of Medicine, Albert Einstein College of Medicine, Bronx, NY, USA; 4 Department of Pathology, School of Medicine, University of Washington, Seattle, WA, USA; 5 Baptist Memorial Medical Education, Memphis, TN, USA

## Abstract

Anchorage-independent survival after intravasation of cancer cells from the primary tumor site represents a critical step in metastasis. Here, we reveal new insights into how MUC13-mediated anoikis resistance, coupled with survival of colorectal tumor cells, leads to distant metastasis. We found that MUC13 targets a potent transcriptional coactivator, YAP1, and drives its nuclear translocation via forming a novel survival complex, which in turn augments the levels of pro-survival and metastasis-associated genes. High expression of MUC13 is correlated well with extensive macrometastasis of colon cancer cells with elevated nuclear YAP1 in physiologically relevant whole animal model systems. Interestingly, a positive correlation of MUC13 and YAP1 expression was observed in human colorectal cancer tissues. In brief, the results presented here broaden the significance of MCU13 in cancer metastasis via targeting YAP1 for the first time and provide new avenues for developing novel strategies for targeting cancer metastasis.

## Abstract (precis)

This study reveals that MUC13 plays a critical role in the metastasis-associated processes via interaction with YAP1 and nuclear translocation of the YAP1-mediated survival complex and a biochemical intervention that interferes with MUC13–YAP1 complex formation can help develop new therapeutics.

## Introduction

Colorectal cancer (CRC) is highly prevalent worldwide. It is the second most lethal cancer (both sexes combined) in the United States, according to the American Cancer Society’s 2023 statistics (Siegel et al, 2023). However, the mortality and death rates have decreased in recent years because of increasing changes in lifestyle, prescreening, and advancements in treatments (Siegel et al, 2017). Most sporadic CRCs arise from polypoid adenomas that develop into intramucosal carcinomas (stage 0), eventually transforming into malignant tumors. Thus, early diagnosis and endoscopic excision of early lesions are critical components of CRC care (Mizutani et al, 2020). The overall 5-yr survival rate of CRC drops remarkably if cancer is diagnosed at the metastatic stage compared with nonmetastatic localized disease (Siegel et al, 2017). As metastasis is the predominant cause of cancer-related deaths (Gupta & Massague, 2006), understanding the precise molecular mechanisms of the progression of the localized disease to metastasis at distant sites is vital for reducing cancer mortality.

Metastasis is a complex multistep process; successful metastasis includes escaping tumor cells from the primary tumor and settling at new distant tissues. Loss of anchorage or detachment to the ECM induces programmed cell death (anoikis), and the ability of cells to evade this is referred to as anoikis resistance (Kim et al, 2012). Only a small fraction of detached cells survive and remain viable (about 0.02%) to form a metastatic lesion (Celià-Terrassa & Kang, 2016). Thus, the detachment and escape of cancer cells from the ECM is a critical stage for metastasis (Walker et al, 2018).

Given the complexity of this process, a failure at any step in the metastatic cascade might prevent the formation of metastatic lesions, anoikis resistance being one of the critical steps among them (Fidler, 1990; Gupta & Massague, 2006). The ECM-detached tumor cells must overcome the body’s natural defense against anchorage-independent growth inhibition for survival. These defenses create a significant barrier to metastasis after cells detach from the ECM (Frisch & Francis, 1994; Simpson et al, 2008; Corkery et al, 2018). Anoikis occurs by stimulating two different apoptotic pathways: extrinsic stimulation, such as the FADD pathway, or intrinsic stimulation, leading to the activation of Bax or Bak and the release of cytochrome c (Simpson et al, 2008; Taddei et al, 2012). The process of anoikis has several mechanisms of resistance, such as up-regulation of pro-survival pathways (PI3/AKT, ERK, FAK, IKL, and src tyrosine kinase), transcription factors such as Jun, Fos, and NF-kB (Simpson et al, 2008; Taddei et al, 2012; Paoli et al, 2013), and dysregulated expression of integrins (Corkery et al, 2018). In addition, the epithelial-to-mesenchymal transition process also leads to the development of an inherent anoikis resistance (Taddei et al, 2012; Paoli et al, 2013).

A significant barrier to the successful treatment of metastatic disease involves the acquisition of dormant and chemo-resistant phenotypes by disseminated tumor cells upon their arrival in metastatic niches. These cells can reactivate proliferative programs to establish deadly metastases years to decades after the initial implementation of therapy, making them one of the most clinically relevant targets. Understanding and identifying the pathways during the initial migration of tumor cells from the primary tumor to metastatic sites will help design new therapeutic modalities to treat metastatic disease. The aberrant expression of YAP1 has been identified in CRC and other cancers. YAP1, specifically through the Hippo pathway, governs several critical biological activities that facilitate cell survival, growth, differentiation, and organ size (Mo et al, 2014; Ma et al, 2015; Wierzbicki & Rybarczyk, 2015). YAP1 is a downstream transcription factor in this pathway (Zhao et al, 2012; Shao et al, 2014; Wierzbicki & Rybarczyk, 2015; Haemmerle et al, 2017; Sanchez-Vega et al, 2018). Under normal conditions, the Hippo–YAP1 signaling axis plays a vital role in cell homeostasis within the colon, whereas YAP1 is up-regulated in colorectal tumors (Wang et al, 2013). Up-regulation of YAP1 has been associated with increased cell proliferation, survival, drug resistance, epithelial-to-mesenchymal transition activation, and anchorage-independent growth (Ma et al, 2015; Wierzbicki & Rybarczyk, 2015). In addition, β-catenin is a transcription factor that modulates the Wnt signaling pathway, playing a critical role in the development of CRC (Herbst et al, 2014; Krausova & Korinek, 2014; Basu et al, 2016). Studies have suggested a cooperativity between YAP1 and β-catenin in cancer cell survival via up-regulation of antiapoptotic markers such as BIRC5 and the Bcl2 family of proteins (Konsavage et al, 2012; Rosenbluh et al, 2012; Bernascone & Martin-Belmonte, 2013; Krausova & Korinek, 2014; Yang et al, 2017; Sanchez-Vega et al, 2018).

MUC13, a transmembrane glycoprotein, has exhibited oncogenic functions in various cancers, including CRC (Gupta et al, 2014). MUC13 comprises of three EGF-like domains, a tandem repeat domain, and a SEA domain within the extracellular domain. MUC13 also has a transmembrane domain and a functional cytoplasmic tail (CT). The MUC13 CT contains several possible phosphorylation sites (Chauhan et al, 2009; Chauhan et al, 2012; Gupta et al, 2014; Sheng et al, 2017b). In a recent study from our laboratory, MUC13 was shown to functionally interact with and activate HER2 (p1248 site), stimulating pro-survival signaling pathways such as ERK1/2, AKT, FAK, and PAK1. It also induced glucose metabolism through activation/nuclear translocation of NF-κB p65 and phosphorylation of IκB to promote tumorigenic features in pancreatic cancer (Khan et al, 2017; Kumari et al, 2018). MUC13 expression has been correlated with cancer progression, disease outcome, patient’s poor prognosis, and biophysical changes in cancer cells (Khan et al, 2018; Massey et al, 2020). In accordance with our studies, others have also demonstrated the tumorigenic roles of MUC13 by modulating various signaling pathways (Shimamura et al, 2005; Walsh et al, 2007; Sheng et al, 2011; Sheng et al, 2013; Sheng et al, 2017a; Sheng et al, 2017b; Mito et al, 2018; Sheng et al, 2019). However, its involvement in cancer metastasis remains elusive. For the first time, we discovered in this study the critical role of MUC13 in cancer metastasis. Our studies describe how MUC13 facilitates metastasis after disseminating tumor cells from the primary tumor site by targeting YAP1 and influencing its nuclear translocation, followed by the activation/expression of pro-survival/metastasis-associated genes. This study has found that the MUC13 mucin is a key molecular player that accords anchorage-independent survival and facilitates metastasis processes by targeting the Hippo pathway modulator, YAP1, a potent transcriptional coactivator. This novel MUC13-YAP1–driven molecular mechanism provides a crucial survival advantage to anchorage-independent circulating tumor cells for successful extravasation, homing, and aggressive cancer metastasis to occur at new distant sites.

## Results

### MUC13 expression is induced during anchorage-independent survival

**Figure 1.**
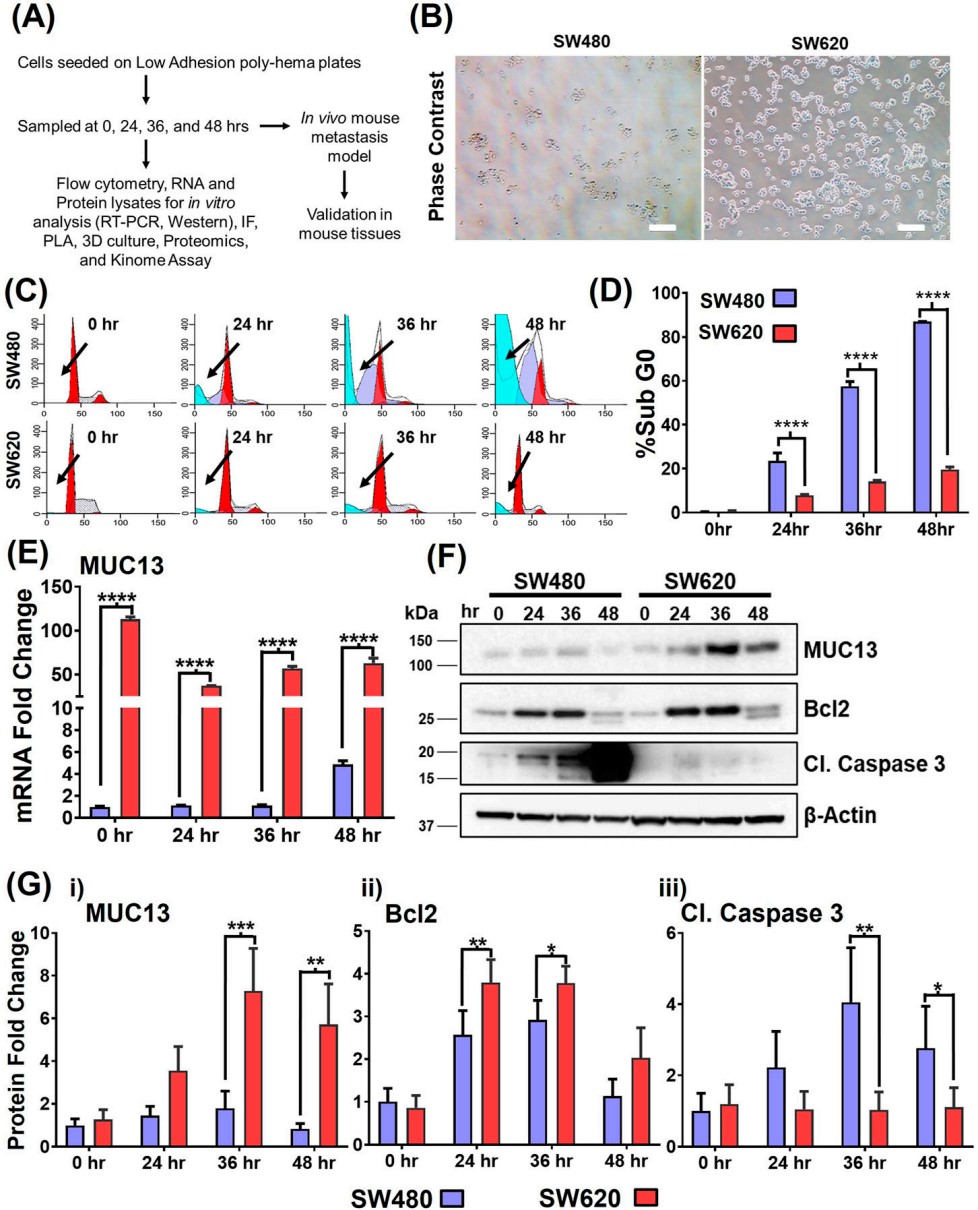
Higher MUC13 expression is associated with better anchorage-independent survival in isogenic cancer cells. **(A)** Outline of the model and strategy adopted for the proposed study. **(B)** Survival of two isogenic CRC cells with high (SW620) and low (SW480) MUC13 expression after anchorage-independent stimulation. Cells plated on low adhesion (poly-HEMA-coated) culture dishes for 36 h were collected and re-plated on regular culture dishes and photographed under a phase contrast microscope after 8 h (Scale bar, 20 μm). **(C, D)** Cell cycle analysis of SW480 and SW620 at 0, 24, 36, and 48 h after anchorage-independent cell survival or anoikis stimulation. **(C)** The histogram represents the cell cycle distribution, and the arrows indicate the location of the Sub G0 population of SW480 and SW620 cells. Quantification (D) of % Sub G0 population of SW480 and SW620 cells at different time points. Unpaired *t* test, n = 3. Data are represented as mean ± SEM. *P < 0.05, **P < 0.01, and ***P < 0.001 denotes significant differences among tested cells. **(E, F, G)** Cells (SW480 and SW620) were subjected to anchorage-independent stimulation for different time points (0, 24, 36, and 48 h) and subjected to MUC13 mRNA analysis by qRT–PCR (E) and Western blot analysis (F). Quantification (G) of SW480 and SW620 immunoblots and qRT–PCR at 0, 24, 36, and 48 h after anchorage-independent stimulation. The data are shown as mean ± SEM in all panels. Sidak’s multiple comparison tests are used after a two-way ANOVA. *P < 0.05, **P < 0.01, ***P < 0.001, and ****P < 0.0001 denote significant differences. Data are representative of at least three individual experiments.

We established and optimized an anoikis induction model to study an essential step in which cancer cells disseminate from the primary tumor and navigate anchorage-independent survival to finally reattach at distant sites. Two isogenic CRC cell lines, SW480 and SW620, isolated from the same patient’s primary tumor site and metastatic tumor site, respectively, were used for the studies. Fig 1A outlines the model and strategy of the study performed. In this anchorage-independent anoikis model, SW620 cells showed better survival than SW480 cells. SW620 cells reseeded on standard tissue culture dishes (after 36 h of incubation on low-adhesion plates) showed increased cell viability and attachment capability (Fig 1B). In cell cycle analysis, the subG0 population peak was much smaller in SW620 cells compared with SW480 cells, whereas SW480 cells exhibited a larger subG0 population compared with metastatic SW620 cells (Fig 1C and D). The comparative expressions of MUC13, along with survival marker protein (Bcl2) and endpoint apoptosis marker (cleaved caspase 3), were examined in SW480 and SW620 cells in an anchorage-independent induction assay as described earlier (Frisch & Francis, 1994; Simpson et al, 2008; Corkery et al, 2018). A pronounced increase in the expression of MUC13 and Bcl2 was observed in SW680 cells at 24–36 h (fourfold increase) compared with 0 h, whereas SW480 cells showed only a moderate (twofold) increase in the expression of these proteins compared with the 0 h expression level.

In contrast, SW480 cells demonstrated a pronounced increase in cleaved caspase expression at 24, 36, and 48 h compared with its 0 h expression level, whereas no apparent change was observed in SW620 cells (Fig 1E–G). MUC13 has been shown to be oncogenic and plays a role in activating key survival pathways in cancer (Chauhan et al, 2012; Gupta et al, 2014; Khan et al, 2017; Sheng et al, 2017b). We determined a pattern of MUC13 expression in SW480 and SW620 cells in a time-dependent manner in the anchorage-independent survival model. Interestingly, MUC13 exhibited a cyclic expression pattern among SW480 and SW620 cell lines with peak expression at 36 h at the protein level. The spike of MUC13 expression at 36 h was more pronounced in SW620 cells (Fig 1E–G). These data suggest that MUC13 plays an important role in the survival of cancer cells during anchorage-independent conditions and the development of anoikis resistance in the circulation time.

### MUC13 promotes survival and spheroid formation of cancer cells

**Figure S1.**
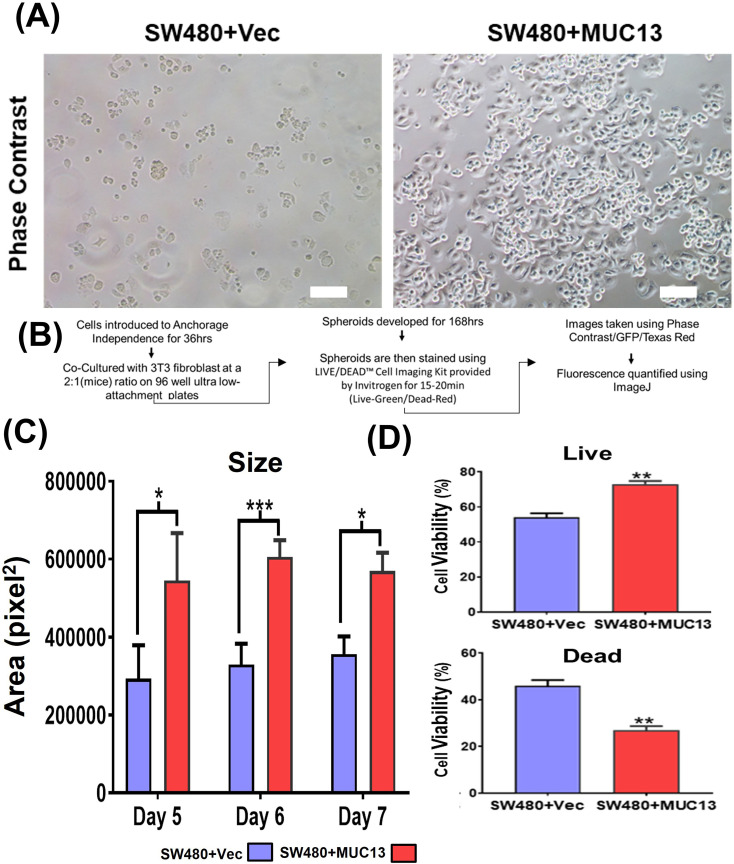
MUC13 expression promotes anchorage-independent survival of cancer cells. **(A)** Phase contrast images of MUC13 overexpression cells (SW480+Vec and SW480+MUC13 cells) after anchorage-independent growth (36 h) and re-plating on regular culture dishes. After 8 h, cells were gently washed (PBS) and photographed under a phase contrast microscope (scale bar, 20 μm). The images depict at least three independent experiments. **(B)** Schematic of 3D culture assay. **(C)** Quantification and size analysis of spheroids at days 5, 6, and 7. Unpaired *t* test, n = 3. Data are represented as mean ± SEM. *P < 0.05, **P < 0.01, and ***P < 0.001 denote significant differences. **(D)** Quantification of % live and dead cells in spheroids generated from SW480+Vector and SW480+MUC13 at day 7 after anchorage-independent stimulation. Unpaired *t* test, n = 3. Data are represented as mean ± SEM. *P < 0.05, **P < 0.01, and ***P < 0.001 denote significant differences. In all panels, the data are representative of at least three individual experiments, and data are represented as mean ± SEM. *P < 0.05, **P < 0.01, and ***P < 0.001 denote significant differences.

**Figure 2.**
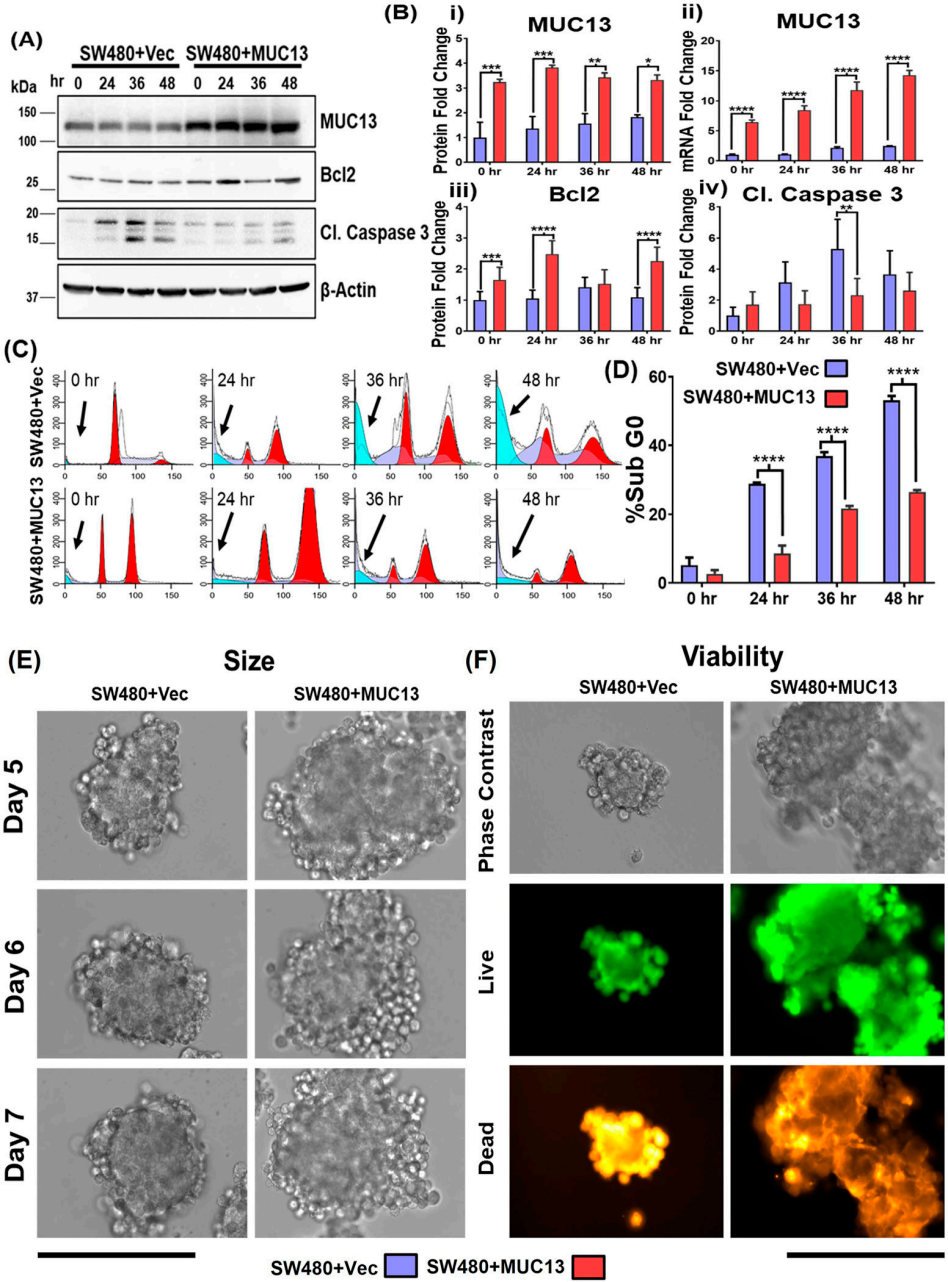
Ectopic MUC13 expression enhances anchorage-independent survival of cancer cells. **(A, B)** Western blot analysis of MUC13 overexpressing (SW480+MUC13) and control (SW480+Vec) cell lines at the indicated time points after anchorage-independent stimulation. Quantification (B) of the immunoblots for indicated proteins and qRT–PCR analysis of MUC13 at different anoikis-induction time points. The data are represented as mean ± SEM. Sidak’s multiple comparison tests are used after a two-way ANOVA. **P < 0.01, ***P < 0.001, and ****P < 0.0001 denote significant differences. **(C, D)** Cell cycle analysis of SW480+Vector and SW480+MUC13 cells at 0, 24, 36, and 48 h after anchorage-independent stimulation. Histogram represents the cell cycle distribution, and arrows indicate the location of the Sub G0 population (C), Quantification (D) of the % Sub G0 population of SW480+Vector and SW480+MUC13 at different time points. Data are represented as mean ± SEM. Sidak’s multiple comparison tests are used after a two-way ANOVA. *P < 0.05, **P < 0.01, ***P < 0.001, and ****P < 0.0001 denote significant differences. **(E)** Representative phase contrast images of spheroids generated from SW480+Vector and SW480+MUC13 after anchorage-independent stimulation (scale bar, 100 μm). **(F)** Cell viability analysis shows representative phase contrast and fluorescence images of live (green) and dead (red) stains (scale bar, 100 μm).

To elucidate the significance of MUC13 in developing anchorage-independent survival of cancer cells, stable MUC13 overexpressing (SW480+MUC13) and MUC13 knockdown (SW620+shMUC13) cell lines were developed. After a 36-h incubation on low attachment poly-Hema plates, MUC13 overexpressed SW480 cells (SW480+MUC13) were reattached at a higher density than that of vector control (SW480+Vec) to regular tissue culture plates (Fig S1A). MUC13 mRNA expression in the SW480+MUC13 cell line was higher and gradually increased during 0–36 h time points (Fig 2Bii). The expression of the antiapoptotic protein Bcl2 showed a significant increase in SW480+MUC13 cells at 24 and 48 h, along with MUC13 expression as compared with the vector-only control. The overexpression of MUC13 led to the decrease of cleavage of caspase 3 in SW480 cells. MUC13 expression was constantly increased in SW480+MUC13 cells from 0 to 48 h, whereas it was either reduced or maintained in vector control cells (Fig 2A and B). As a result, SW480+MUC13 cells showed much lower (30%) apoptotic cell death as compared with SW480+Vec cells (50%) during anoikis induction at 48 h (Fig 2C and D). To mimic an in vitro micrometastasis model, these cells were grown in a 3D cell culture model for five days using mouse fibroblast 3T3 cells after induction of anchorage-independent survival for 36 h, as shown in Fig S1B. In this model, SW480+MUC13 cells generated larger spheroids than SW480+Vec cells. SW480+MUC13-generated spheroids continued to increase in size from day 5 to day 7, whereas the size of SW480+Vec spheroids remained similar over time (Figs 2E and S1C). Next, we evaluated the ratio of live and dead cells in the spheroids generated from SW480+MUC13 and SW480+Vec cells using the LIVE/DEAD Cell Imaging kit. In this assay, SW480+MUC13 spheroids showed a higher percentage (73% ± 3%) of the live cell population (green fluorescence) compared with SW480+Vec (53% ± 4%) cells (Figs 2F and S1D).

**Figure 3.**
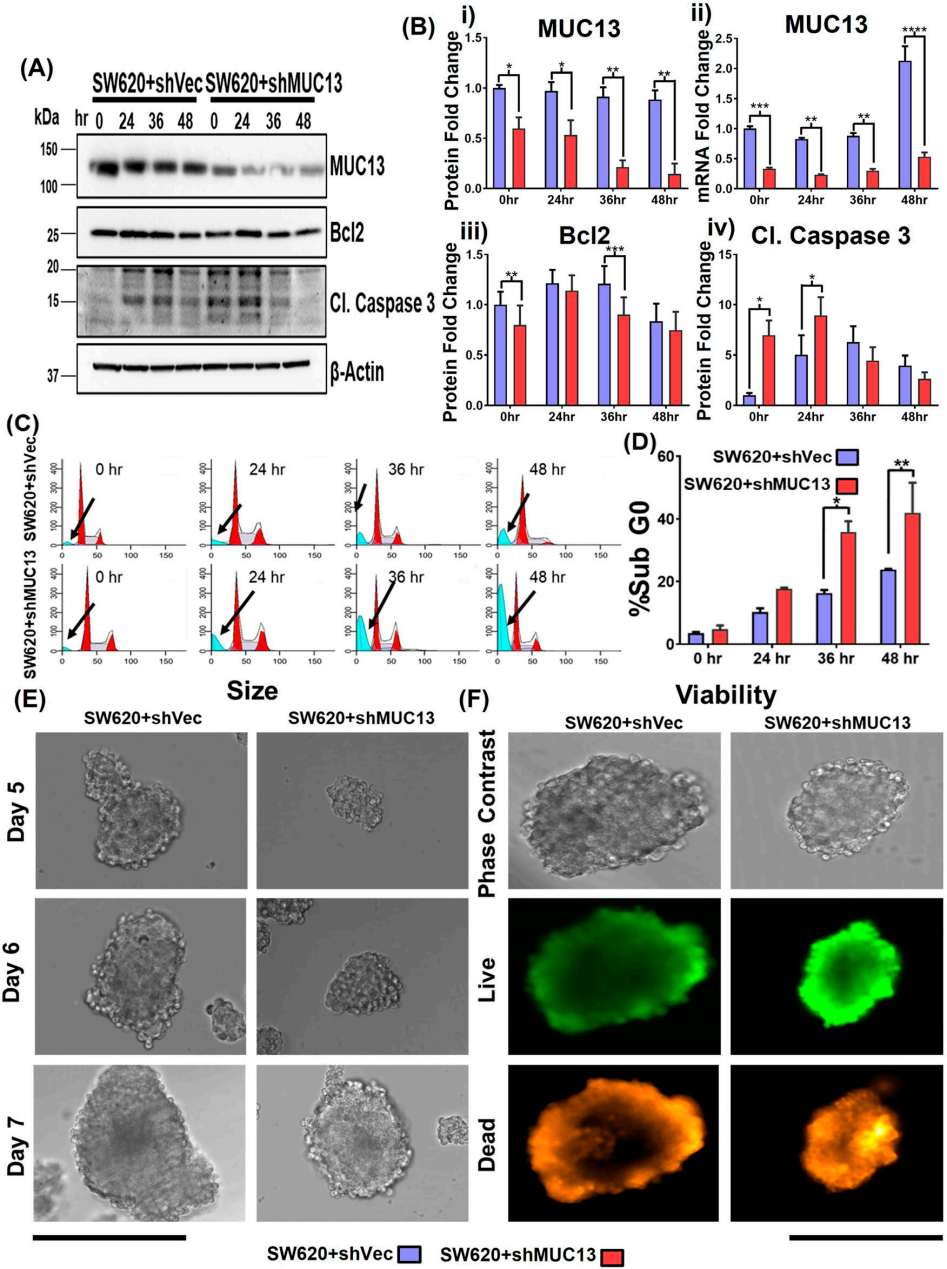
MUC13 knockdown decreases anchorage-independent survival of cancer cells. **(A)** Western blot analysis of MUC13 knockdown (SW620+shMUC13) and its respective control (SW620+shVec) cell lines after anchorage-independent stimulation. **(B)** Quantification of immunoblots at indicated time points after anchorage-independent stimulation and qRT–PCR analysis of MUC13 at different anoikis-induction time points. Data are represented as mean ± SEM. Two-way ANOVA followed by Sidak’s multiple comparison test. **(C, D)** Cell cycle analysis (C) and quantification (D) of Sub G0 population of cells at different time points after anchorage-independent stimulation. All panels: The data are representative of at least three individual experiments. The data are shown as mean ± SEM. Sidak’s multiple comparison test is used after a two-way ANOVA. *P < 0.05, **P < 0.01, ***P < 0.001, and ****P < 0.0001 denote significance. **(E)** Representative phase contrast images of spheroid generated from MUC13 knockdown (SW620+shMUC13) and control (SW620+shVector) cells after anchorage-independent stimulation (Scale bar, 100 μm). **(F)** Representative phase contrast and fluorescence images of cell viability in spheroids and quantification of live (green)/dead (red) cells at day 7 after anchorage-independent stimulation (scale bar, 100 μm).

**Figure S2.**
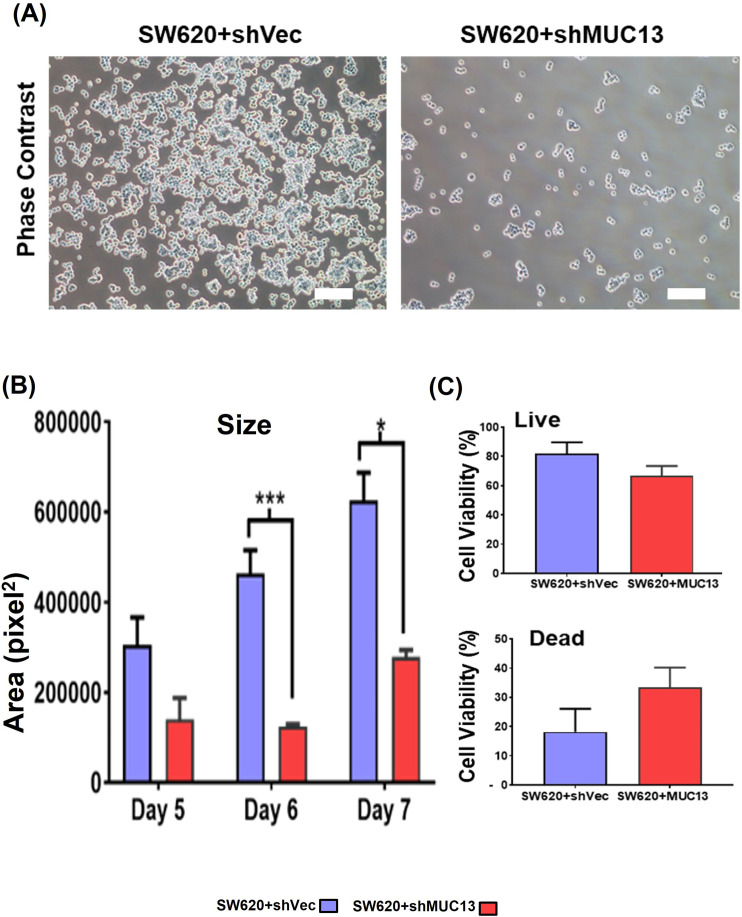
Repression of MUC13 inhibits anchorage-independent survival of cancer cells. **(A)** Phase contrast images of the MUC13 knockdown cells (SW620+shCtrl and SW620+shMUC13 cells) after anchorage-independent growth (36 h) and re-plating on regular culture dishes. After 8 h, the cells were gently rinsed with PBS and imaged using a phase contrast microscope (scale bar, 20 μm). The images depict at least three independent experiments. **(B)** Quantification and size analysis of spheroids at days 5, 6, and 7. Unpaired *t* test, n = 3. Data are represented as mean ± SEM. *P < 0.05, **P < 0.01, and ***P < 0.001 denote significant differences. **(C)** Quantification of % live and dead cells in spheroids generated from SW620+shCtrl and SW620+shMUC13 at day 7 after anchorage-independent stimulation. Unpaired *t* test, n = 3. Data are represented as mean ± SEM. *P < 0.05, **P < 0.01, and ***P < 0.001 denote significant differences. In all panels, the data are representative of at least three individual experiments, and data represented as mean ± SEM. *P < 0.05, **P < 0.01, and ***P < 0.001 denote significant differences.

The MUC13 knockdown cell line (SW620+shMUC13), along with the control (SW620+shCtrl), confirmed the role of MUC13 in anoikis resistance. Unlike the overexpression, MUC13 knockdown in SW620 cells reversed the observed molecular, cellular, and functional effects. After 36 h of incubation (on low attachment poly-Hema plates), lower reattachment was observed in MUC13 knockdown SW620 cells and lowered MUC13 mRNA (Figs 3Bii and S2A). Similarly, Bcl2 and MUC13 showed a significant decrease in expression, whereas cleaved caspase 3 increased expression in MUC13 knockdown cells (Fig 3A–D). Knockdown of MUC13 also decreased the 3D spheroid size and increased the ratio of dead cells (Figs 3E and F and S2B and C). These results clearly suggest that MUC13 expression influences the survival of cancer cells under anchorage-independent conditions and helps cancer cells to resist the anoikis process.

### MUC13 forms a molecular complex with YAP1 and alters YAP1 expression/subcellular localization

**Figure 4.**
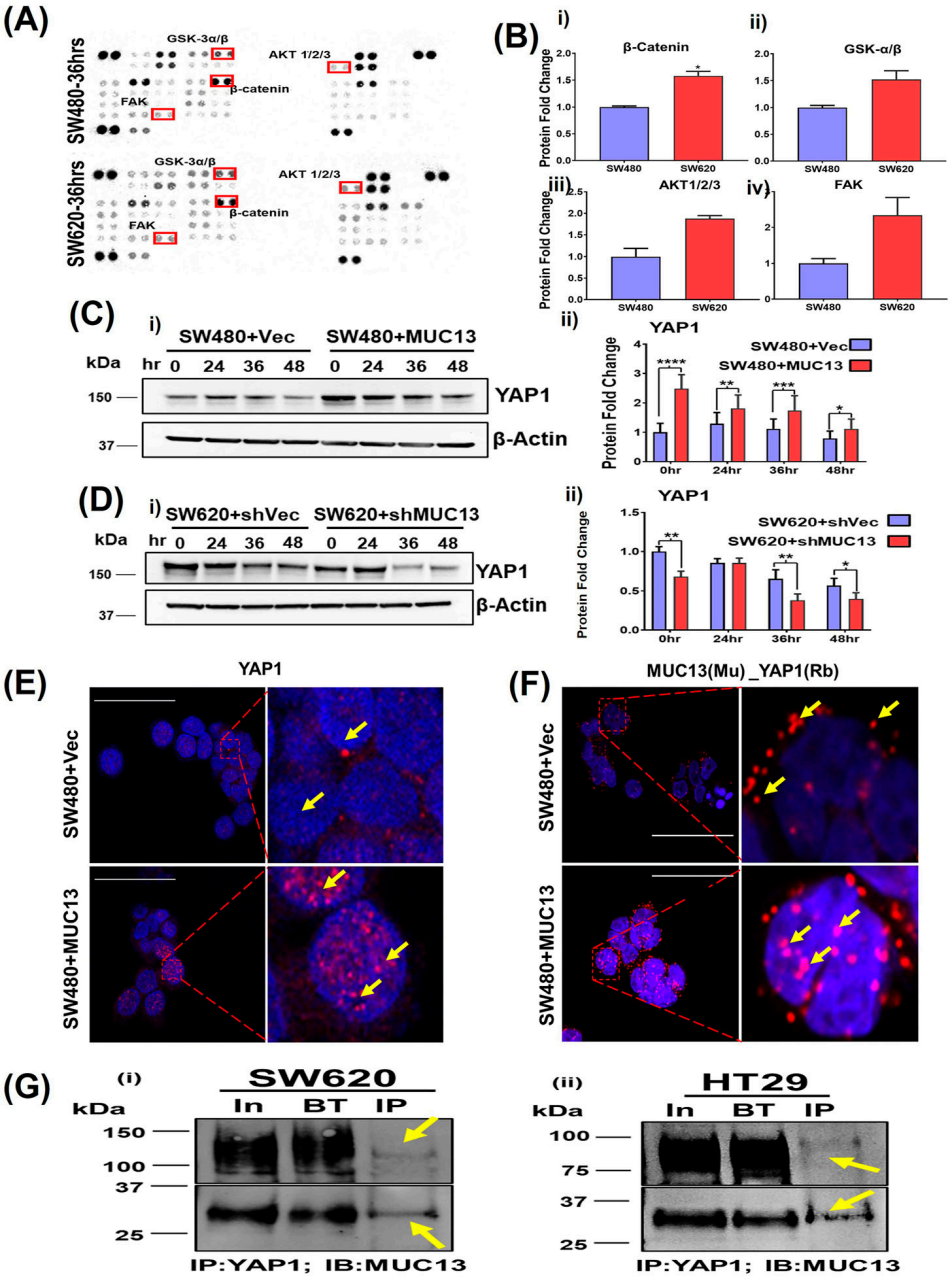
MUC13 and YAP1 proteins colocalize and interact during anoikis-induced conditions. **(A)** Human kinase array analysis with the high (SW620) and low (SW480) MUC13-expressing isogenic cell lines after 36 h of anchorage-independent stimulation. The box indicates the differential expression of kinases with increased (red) and decreased (blue) expression. **(B)** Quantitation of the protein fold change of total β-catenin, GSK3-α/β (S21/S9), AKT1/2/3(T308), and FAK(Y397) phosphorylation. **(C, D)** Immunoblot analysis of YAP1 protein using MUC13 overexpressing knockdown cells. **(C, D)** Quantification of YAP1 expression in (C) SW480+Vector and SW480+MUC13, and (D) SW620+shVector and SW620+shMUC13. Data are representative of at least three individual experiments. The data are shown as mean ± SEM. Sidak’s multiple comparison test is used after a two-way ANOVA. *P < 0.05, **P < 0.01, ***P < 0.001, and ****P < 0.0001 denote significant differences. MUC13 overexpressing and vector control stable cell lines 36 h post anoikis induction, pelleted, cryofixed, and cryosectioned on slides. **(E)** Confocal imaging using YAP1 antibody in MUC13 overexpressing show higher YAP1 localization in the nucleus compared with vector-only control (scale bar, 50 μm). **(F)** PLA was performed using indicated anti-mouse (MAb) and anti-rabbit (PAb) antibodies along with PLA probes. Red dots indicated by yellow arrows represent the physical interaction among listed proteins in used cell lines. MUC13 expression influenced the interaction of YAP1 along with their nuclear translocation. The images depict at least three independent experiments (scale bar, 50 μm). **(G)** (i, ii) YAP1 and MUC13 physically interact in CRC cells. Immunoprecipitation of YAP1 in SW620 (i) and HT29 (ii) cells were able to pull down MUC13, as evidenced by immunoblot for MUC13 with I.P. of YAP1. Two different fractionated forms of MUC13 are indicated.

SW480 and SW620 are isogenic cell lines, and in the present anoikis model, they have shown differential metastatic potential, which is correlated with MUC13 expression. To identify the signaling pathways associated with MUC13’s role in cell survival and anoikis resistance, we performed Proteome Profiler Kinome Array (R & D Human Kinome array profiler) analysis using SW480 and SW620 cells that were incubated on low attachment poly-Hema plates for 36 h. In Kinome array analysis, SW620 cells have shown relatively higher expression of some anoikis resistance-associated known anti-apoptotic signatures such as pFAK^Y397^, pAKT^T308^, and phosphorylated GSK-3α/β (S21/S9), which is a key regulator of β-catenin (Taddei et al, 2012; Cai et al, 2015). In addition, we observed higher expression of total β-catenin in SW620 (1.57-fold increase) cells as compared with SW480 at 36 h (Fig 4A and B). Furthermore, several other kinases were differentially expressed (up-regulated or down-regulated) in these two cell lines (Table S1).

Table S1. Anchorage-independent survival-associated kinases. List of 12 kinases modulated during anchorage-independent stimulation at 36 h. There is an increase in pro-survival kinase AKT and FAK, an increase in total β-catenin, and an increase in phosphorylation of GSK3-α/β and p53.

**Figure S3.**
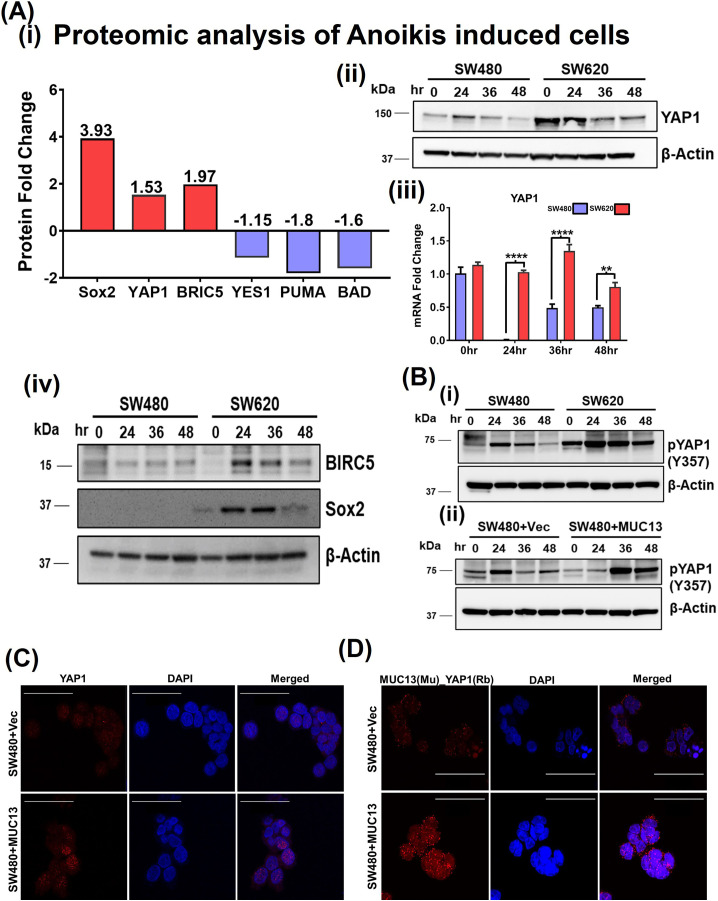
Influence of MUC13 on proteome and YAP1 phosporelation/subcellular localization. **(A)** (i, ii, iii) Key proteins involved in anchorage-independent survival. Proteomic analysis (iTRAQ/P.M.T.) of anoikis-induced cells (SW480 and SW620) at 36 h. Fold changes of the key proteins are shown (i). Immunoblot analysis of YAP1 protein to validate the result of proteomic analysis using isogenic cells SW480 and SW620 (ii), RTPCR analysis of YAP1 mRNA to show YAP1 mRNA expression precedes the translation event (iii). Immunoblot analysis of BIRC5 and SOX2 protein to validate the result of proteomic analysis (iv). **(B)** (i, ii) Immunoblot analysis of pYAP1 (Y357) in isogenic cell lines SW480 and SW620, and MUC13 overexpression SW480+Vec and SW480+MUC13. **(C)** Expanded panels of IF analysis of YAP1 in SW480+Vec and SW480+MUC13 from Fig 4E (scale bar, 50 μm). **(D)** Expanded panels of PLA analysis of MUC13_YAP1 in SW480+Vec and SW480+MCU13 form Fig 4F (scale bar, 50 μm).

Quantitative mass spectrometry analyses (iTRAQ) of these two isogenic cell lines after induction of anchorage-independent survival conditions were performed to further understand the differential protein profile. Proteomic analysis of anchorage-independent stimulated SW480 (low MUC13 expressing) and SW620 (high MUC13 expressing) showed remarkable changes in the expression of key YAP1-related survival proteins (BRIC5; 1.97-fold increase, SOX2 3.93-fold increase) and decreased expression of apoptotic proteins (PUMA, 1.8-fold decrease, and BAD, 1.6-fold decrease). This proteomic analysis also showed a notable increase in a key survival and metastasis-associated protein, YAP1, (1.53-fold). The proteomic findings for YAP1 expression were further validated via immunoblotting and qRT–PCR, which showed an increased expression in SW620 cells at 24, 36, and 48 h for YAP1, BRIC5, and SOX2. We also verified YAP1 mRNA by qRT–PCR to make sure transcription precedes translations. (Fig S3Ai–iv). Thus, we sought to investigate the impact of MUC13 expression on YAP1. Our immunoblot analyses suggest influence of MUC13 expression on YAP1 expression as MUC13 overexpression and MUC13 knockdown clearly altered YAP1 expression at indicated time-points in SW480+MUC13 and SW620+shMUC13 cells compared with their respective controls in the anoikis model (Fig 4C and D). YAP1 has different phosphorylation sites; S127 is the most common among them. However, S127 phosphorylation was found to have no relation with the YAP1/β-catenin survival complex (Rosenbluh et al, 2012), and in the same study, Y357 has been shown as a relevant phosphorylation of the survival complex in CRC. Thus, we verified the phosphorylation of YAP1 at Y357 in our anoikis model. After anoikis induction, we observed a high Y357 expression in SW620 and SW480+MUC13 cells. Herein, we observed an association between MUC13 expression and YAP1 phosphorylation at Y357 (Fig S3Bi–ii).

**Figure S4.**
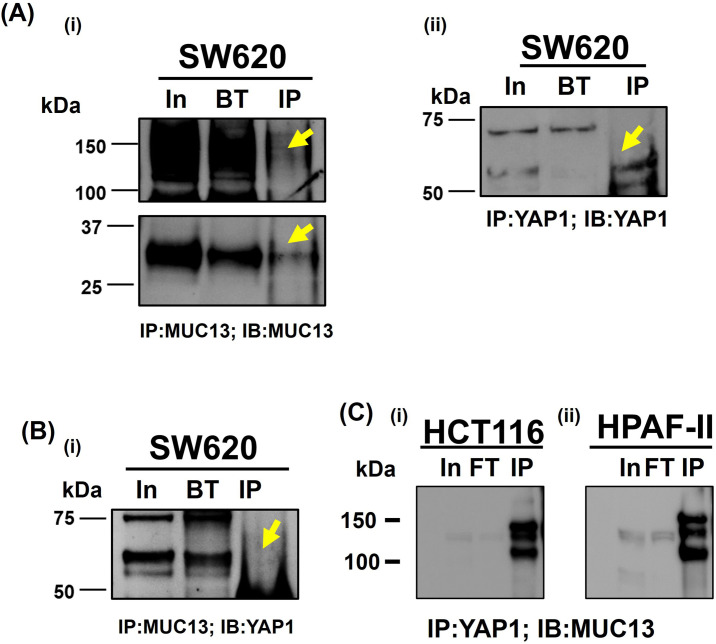
Immunoprecipitation of MUC13 and YAP1 in cancer cells. **(A)** (i, ii) Validation of anti-YAP1 antibody for immunoprecipitation. **(B)** (i) Validation of MUC13 antibody for immunoprecipitation in SW620 cells. **(C)** Immunoprecipitation of YAP1 in HCT116 (i) and HPAF-II (ii).

**Figure 5.**
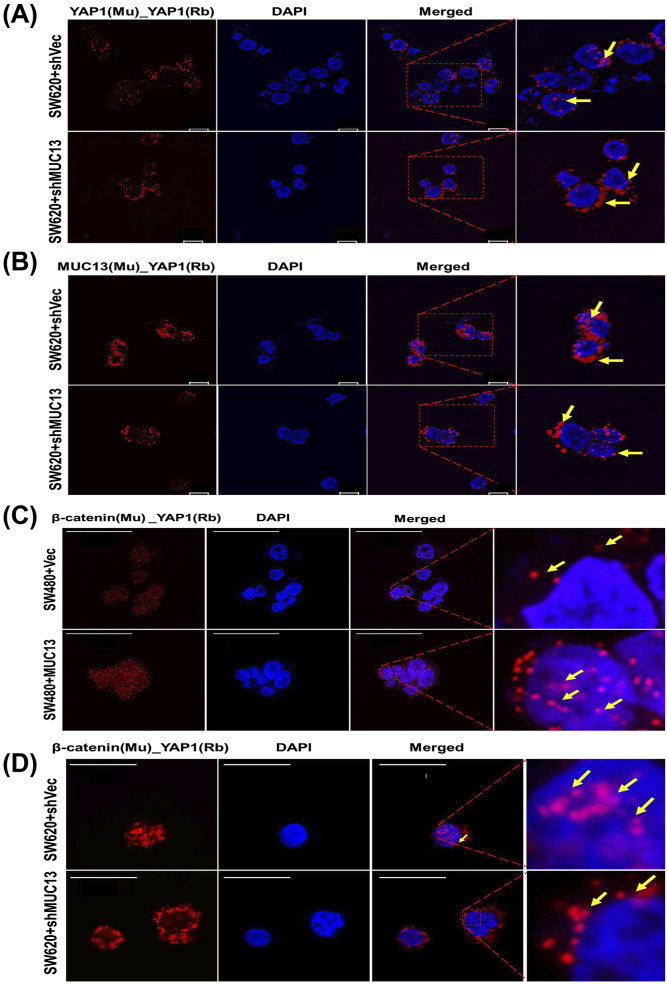
MUC13 facilitates nuclear translocation of YAP1/β-catenin survival complex PLA was performed on pelleted, cryofixed, cryosection cells using the indicated anti-mouse (MAb) and anti-rabbit (PAb) antibodies and PLA probes. Red dots indicated by yellow arrows represent the physical interaction among listed proteins in used cell lines. **(A)** PLA analysis of YAP1 in MUC13 knockdown cells (SW620+shMUC13), leading to decreased nuclear localization of YAP1 compared with the ShCtrl cells (scale bar, 10 μm). **(B)** PLA analysis of MUC13 and YAP1 in MUC13 knockdown cells (SW620+shMUC13) showed increased localization to the perinucleus compared with vector control (scale bar, 10 μm). **(C)** Confocal images show higher YAP1/β-catenin survival complexes in MUC13 overexpression (SW480+MUC13) cells compared to vector only. Most of the YAP1/β-catenin complexes are translocated to the nucleus (scale bar, 50 μm). **(D)** Knockdown of MUC13 prevented the nuclear translocation of the YAP1/β-catenin complex as it was predominantly localized outside the nuclear envelope in SW620+shMUC13 cells (scale bar, 20 μm). The images depict at least three independent experiments.

The overexpression of MUC13 also influenced the subcellular localization of YAP1. To determine subcellular localization, cells (SW480+MUC13 and SW480+Vec) were cultured in an anchorage-independent environment for 36 h, cryofixed, sectioned onto slides, and processed for confocal microscopy. An enhanced nuclear localization/expression of YAP1 was observed in MUC13 overexpressing (SW480+MUC13) cells compared with their respective control cells (SW480−Vec) (Figs 4E and S3C and D). These data indicate the influence of MUC13 on YAP1 expression and localization. To determine if MUC13 and YAP1 colocalize, interact, and remain in close molecular proximity during anoikis resistance, a proximity ligation assay (PLA) was performed in the MUC13 overexpressing cell line model (SW480+MUC13), using specific antibodies. This assay combined antibody–oligo conjugates, enzymatic ligation, PCR amplification, and a fluorescent-detection method. This method detects in situ protein–protein proximity/interaction in cells and tissues. For our experimental procedure, in which a few cells survived during the anoikis model, PLA was the most effective and suitable method for the detection of the protein–protein interaction. The interaction between two proteins was observed in this study utilizing the corresponding two primary antibodies produced in different species. The species-specific secondary antibodies (PLA probes) are bound to the primary antibodies, each having a distinct short DNA strand linked to them. When the PLA probes were in molecular proximity (<40 nm), the DNA strands might connect by inserting two additional circle-forming DNA oligonucleotides. The interaction of two functional protein molecules was detected by a staining dot (red dot in our experiment) Fig 4F. In this assay, MUC13 showed molecular proximity to YAP1. However, MUC13 overexpressing cells (SW480+MUC13) showed pronounced interaction between MUC13 and YAP1 in the cell cytoplasm and the nucleus. This interaction was lower and primarily localized in the cytoplasm in SW480+Vec cells (Fig 4F). Lower magnification images of Fig 4E and F are presented in Fig S3C and D. To reconfirm the physical interaction between MUC13 and YAP1, we performed co-immunoprecipitation (co-IP) assays. YAP1 successfully pulled down MUC13 in SW620 and HT29 (Fig 4Gi and ii). Validation assays of MUC13 IP with MUC13 antibody (Fig S4Ai), YAP1 IP with YAP1 antibody (Fig S4Aii), and reverse co-IP of YAP1 with anti-MUC13 antibody in SW620 cells, and co-IP of MUC13 with anti-YAP1 antibody were performed as shown in Fig S4B and C, to show MUC13 and YAP1 interaction in different cancer cell lines. Additional evidence showed MUC13’s influence on this interaction, as MUC13 knockdown restricted nuclear translocation of YAP1 and it was predominantly localized in peri nuclear space, whereas in SW620+shC control cells, YAP1 was predominantly localized in the nucleus. MUC13 and YAP1 interaction and localization was relatively lesser in MUC13 knockdown cells than the vector cells (Fig 5A and B). These data support the notion that MUC13 physically interacts with YAP1 and influence YAP1 expression and nuclear localization.

### MUC13 promotes nuclear translocation of YAP1–β-catenin survival complex

**Figure S5.**
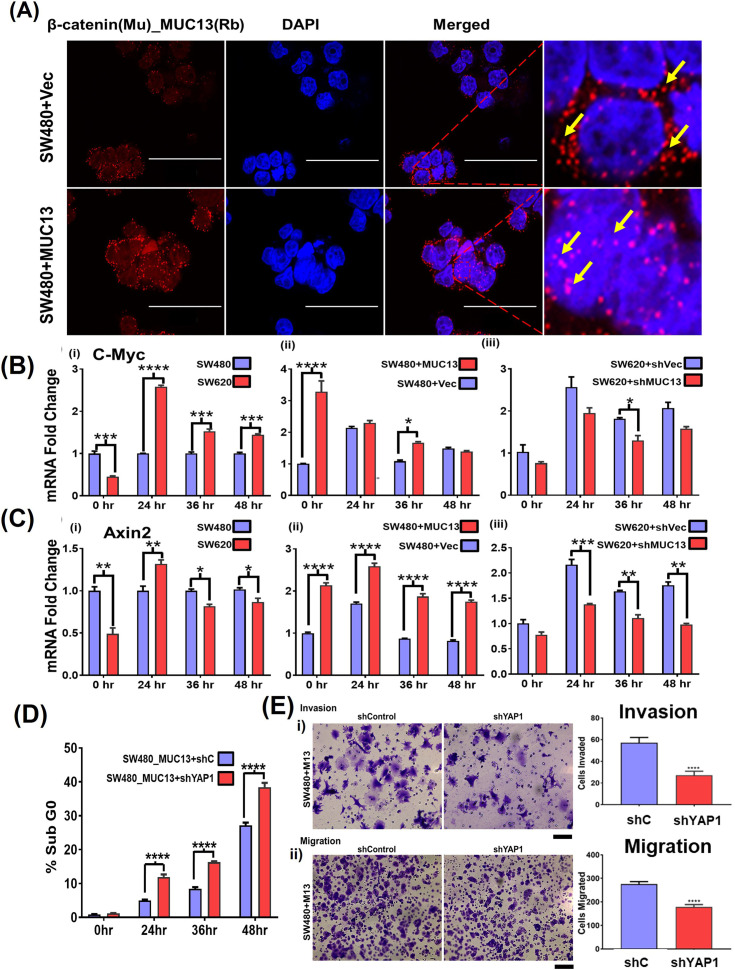
MUC13 facilitates nuclear translocation of downstream oncol YAP1/β-catenigeneicn survival complex PLA was performed on pelleted, cryofixed, cryosection cells using the indicated anti-mouse (MAb) and anti-rabbit (PAb) antibodies and PLA probes. **(A)** β-catenin interaction promotes and (A) PLA analysis of MUC13 and β-catenin in MUC13 overexpressing cells (SW480+MUC13) show higher proximity of these two proteins in the nucleus compared with vector control. The images depict at least three independent experiments (scale bar, 50 μm). **(B, C)** qT–PCR analysis to determine the effect of MUC13 on YAP1/β-catenin survival complex downstream targets genes (C-Myc and Axin 2) in low and high MUC13-expressing isogenic SW480 and SW620, MUC13 overexpression SW480+MUC13 and SW480+Vector, and MUC13 knockdown SW620+shVector and SW620+shMUC13 in (B, C), respectively. YAP1 is required for MUC13-mediated cell survival, migration, and invasion. **(D)** PI-based cell cycle analysis shows knockdown of YAP1 in MUC13 overexpressing cells (SW480_MUC13+shYAP1) increases the sub-G0 population at different time points, indicating the requirement of YAP1 for MUC13-mediated survival function. **(E)** (i, ii) Invasion of knockdown of YAP1 in MUC13 overexpression cell lines and average quantification of the number of cells invaded (i) (scale bar, 50 μm). Migration of knockdown of YAP1 in MUC13 overexpression cell lines and average quantification of the number of cells invaded (ii) (scale bar, 50 μm). Unpaired *t* test, n = 4. Data are represented as mean ± SEM. *P < 0.05, **P < 0.01, and ***P < 0.001 denote significant differences.

Elucidation of cellular and molecular mechanisms by which MUC13 activates YAP1-mediated oncogenic signaling pathways can provide important insights toward cancer metastasis. Recent studies reported MUC13-mediated activation of Wnt/β-catenin signaling in liver cancer (Dai et al, 2018), cross-talk of β-catenin, and Hippo-YAP1 signaling pathways in cancer (Fang et al, 2000; Konsavage et al, 2012; Bernascone & Martin-Belmonte, 2013; Herbst et al, 2014; Sanchez-Vega et al, 2018). In addition, the complexation of YAP1 and β-catenin is reported in cancer cells (Rosenbluh et al, 2012). Thus, we determined the impact of MUC13 on YAP1–β-catenin survival complex formation and its nuclear translocation. For this investigation, we used sections of cryo-fixed MUC13 overexpression (SW480+Vec versus SW480+MUC13) and MUC13 knockdown (SW620+Vec versus SW620+MUC13) cell lines after anoikis induction at 36 h for PLA and confocal analysis. Ectopic MUC13 expression led to a remarkably higher survival complex formation (as determined by co-localization of β-catenin and YAP1) in cells compared with vector control cells. We observed a higher number of PLA dots/localization in the nucleus than in respective vector control cells (Fig 5C). Interestingly, the formation of the YAP1–β-catenin survival complexes was remarkably decreased upon MUC13 knockdown in SW620 cells (SW620+shMUC13). Moreover, MUC13 knockdown effectively restricted nuclear translocation of this survival complex as it was predominantly localized on the periphery of the nucleus as compared with the respective vector control (SW620+shCtrl) cells (Fig 5D). To determine if the translocation of this survival is MUC13-dependent, we performed PLA studies with MUC13–β-catenin in MUC13 overexpressing cells (SW480+MUC13). In this analysis, we observed a higher nuclear localization of the MUC13–β-catenin complex in SW480+MUC13 cells compared with SW480+Vec cells as determined by PLA analysis (Fig S5A).

To further investigate if YAP1 is downstream to MUC13, in YAP1-mediated survival of cancer cells in an anchorage-independent environment, we knockdown YAP1 expression in ectopic MUC13 expressing (SW480+MUC13) cells. The cell cycle analysis shows a significant increase of SW480+MUC13 cells in the sub-G0 phase upon YAP1 knockdown during anchorage-independent growth conditions compared with control (SW480+Vec) cells (Fig S5D). In addition, YAP1 knockdown decreased cellular invasion and migration of SW480+MUC13 cells in the transwell migration and Matrigel invasion assays (Fig S5E). These data clearly suggest that MUC13 expression promotes the formation and nuclear shuttling of the YAP1–β-catenin survival complex to modulate metastasis-associated oncogenic signaling in cancer cells. These findings explain how MUC13 provides anoikis resistance to the surviving cancer cells for successful metastasis and indicate that MUC13 drives its oncogenic function through YAP1-dependent pathways. Dysregulation of the Wnt/β-catenin pathway is a hallmark of CRC (Krausova & Korinek, 2014; Basu et al, 2016); thus, its key downstream target genes (Axin2 and C-Myc) were studied at different time points in our control and with experimental MUC13 expression cell lines (SW480 versus SW620; SW480+Vec versus SW480+MUC13; SW620+Vec versus SW620+MUC13) using our anoikis model by qRT-PCR (Herbst et al, 2014; Dai et al, 2018; Liu et al, 2021). Axin2 and c-Myc showed peak expression at 24 h and maintained higher levels compared with the 0 h time point in SW620 cells, whereas the expression of these proteins remained the same in SW480 cells at all studied time points. MUC13 overexpression in SW480 and MUC13 knockdown SW620 cell lines resulted in the altered expression of C-Myc and Axin 2 (Fig S5B and C).

### MUC13 augments cancer metastasis

**Figure 6.**
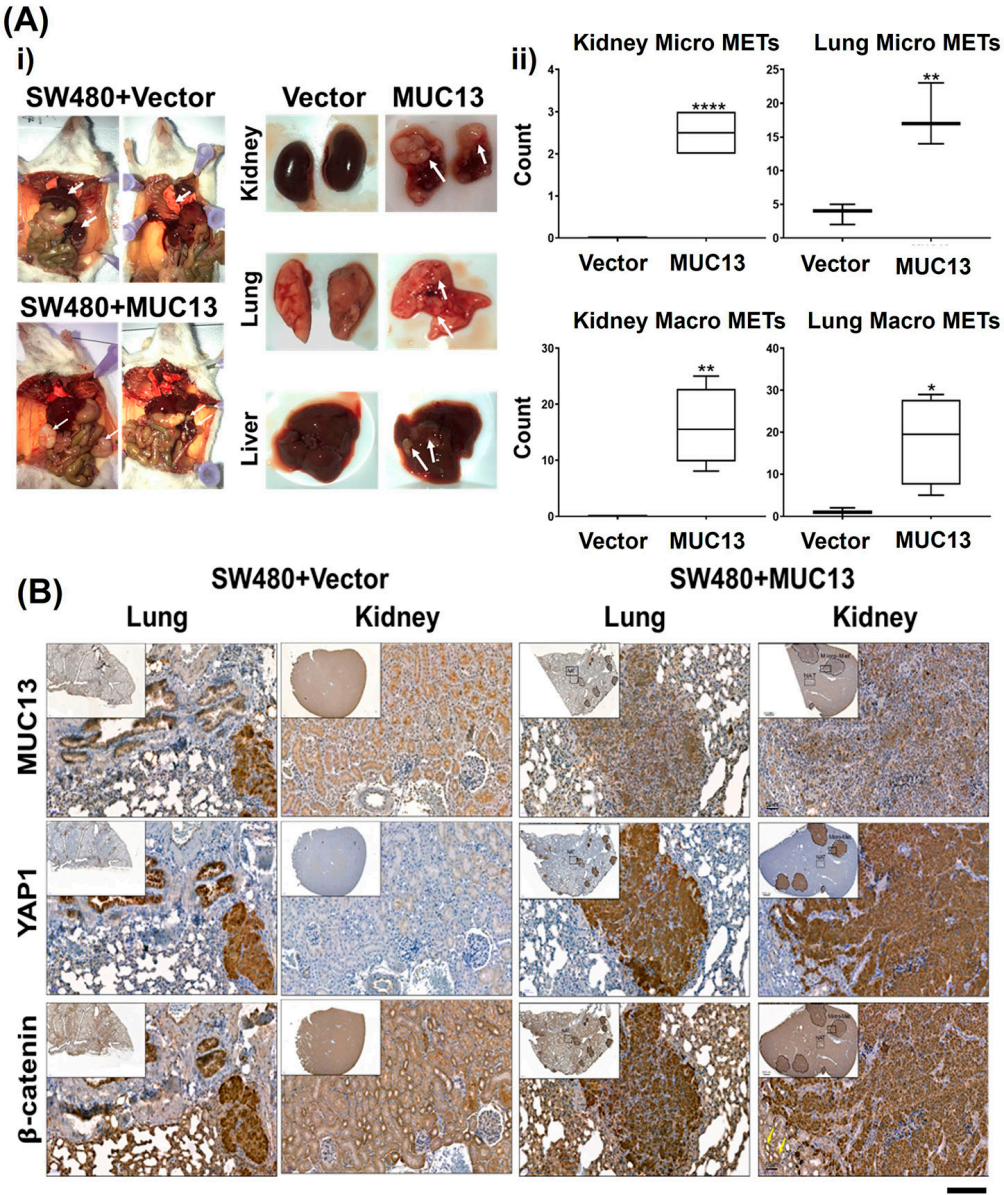
MUC13 enhances metastatic potential of cancer cells. **(A, B)** In vivo metastasis (tail vein) caused by MUC13 overexpressing (SW480+MUC13) and non-overexpressing (SW480+Vector) cells. **(A)** (i, ii) Gross images of representative mouse with metastatic tumors and the three most affected organs—kidney, lung, and liver—are shown (i). MacroMETs and microMETs profiles of mice injected with overexpression of MUC13 compared with mice injected with vector cells in the kidneys and lungs. The data is represented as mean ± SEM. Sidak’s multiple comparison test is used after a two-way ANOVA. *P < 0.05, **P < 0.01, ***P < 0.001, and ****P < 0.0001 denote significant differences (ii). **(B)** IHC analysis in lungs and kidney to demonstrate MUC13, YAP1, and β-catenin protein expression in metastatic lesions (scale bar, 50 μm). MacroMETs and microMETs profile of mice injected with overexpression of MUC13 compared with mice injected with vector cells in the kidneys and lungs.

**Figure S6.**
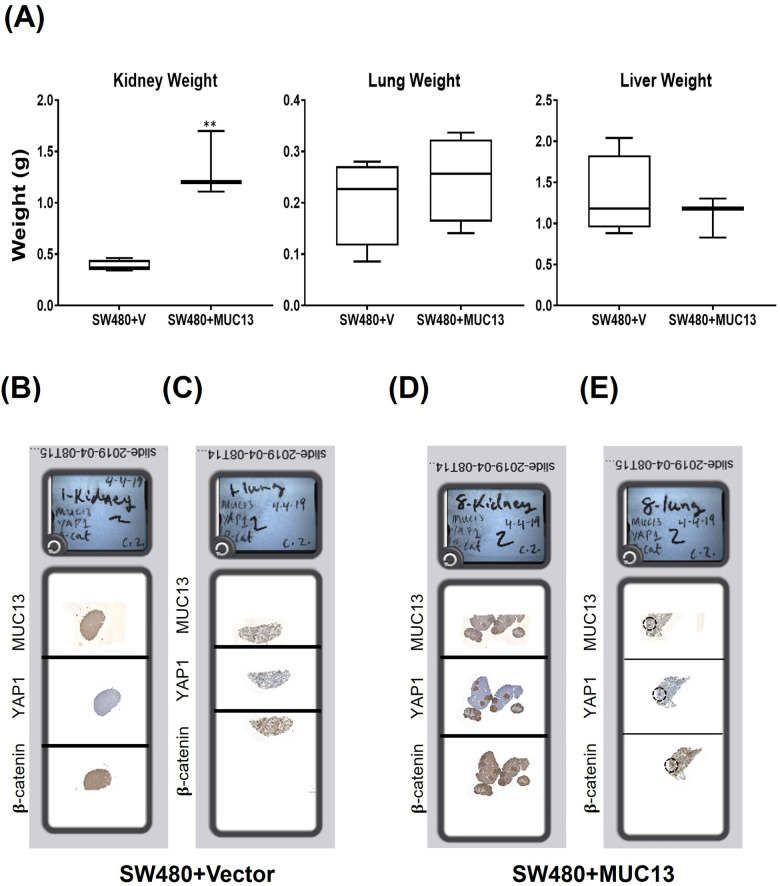
Tail vein metastasis model in NSD mice. **(A)** Wet weight of mice’s three most affected organs, namely the kidney, lungs, and liver, injected with SW480+MUC13 and SW480+Vector cells. Organ weight profile of the kidney, lungs, and liver of mice injected with control and MUC13 overexpressing cells. **(B, C, D, E)** Digitally scanned complete slides of kidney and lung tissues collected from a metastatic mouse model and injected with SW480+Vec (B, C) and SW480+MUC13 (D, E) cells via tail vein. The harvested tissues were stained for MUC13, YAP1, and β-catenin using their respective antibodies. The data are represented as mean ± SEM. Sidak’s multiple comparison test is used after a two-way ANOVA. *P < 0.05, **P < 0.01, ***P < 0.001, and ****P < 0.0001 denote significant differences.

To investigate the impact of MUC13-mediated nuclear translocation of the survival complex (YAP1–β-catenin) on metastatic potential, a tail vein metastatic model was used. After 36 h of anoikis induction, SW480+MUC13 cells, and the control (SW480+Vec) cells were injected into the NSG mice through tail vein injection. The mice were monitored and weighed once a week for 4.5 wk. After 4.5 wk postinjection, the mice were euthanized, and the presence of the metastatic lesions in different organs was analyzed by quantification of the metastatic nodules, as described earlier (Tripathi et al, 2014). The mice injected with MUC13 overexpressing cells (SW480+MUC13) suffered from a significant loss in body weight after 4 wk, whereas there was no loss in body weight of mice injected with vector control cells (SW480+Vec). The lungs and kidneys of the mice injected with MUC13 overexpressing cells weighed more and showed severe metastasis. Although the liver of these mice weighed less, several apparent metastases were observed in the liver. Conversely, mice injected with vector control cells (SW480+Vec) maintained their body weight throughout the study. They showed no apparent metastatic lesions in the kidney, lungs or liver (Figs 6Ai and ii and S6A). To microscopically confirm metastatic lesions and determine the expression/subcellular localization of MUC13, YAP1, and β-catenin, organs were fixed and processed for histology and immunohistochemistry (IHC) analyses. Organs of mice injected with MUC13 overexpressing cells demonstrated several macro/microscopic metastatic lesions along with prominent MUC13, YAP1, and β-catenin staining. Metastatic lesions present in the kidney, lungs, and liver showed considerable nuclear subcellular localization of these proteins along with membrane and cytoplasmic staining (Fig 6B). Mice injected with MUC13 overexpression cells (SW480+MUC13) showed a significantly greater number of macroMETs (>400 μm) and microMETS (<400 μm) in the kidneys and lungs (Tables S2 and S3). The complete slides with the stained tissue are shown in Fig S6B–E. Tables S2 and S3 represent significant pathological features and a mouse-by-mouse breakdown of the difference in the number of macroMETs (>400 μm) and microMETs.

Table S2. IHC staining localization of YAP1, MUC13, and β-catenin in lung and kidney of mouse tissues.

Table S3. Scoring of the number of macro and micro met in the lungs and kidneys of mice along with their IHC scoring.

### Clinical significance of MUC13 and YAP1 co-expression in cancer progression

**Figure S7.**
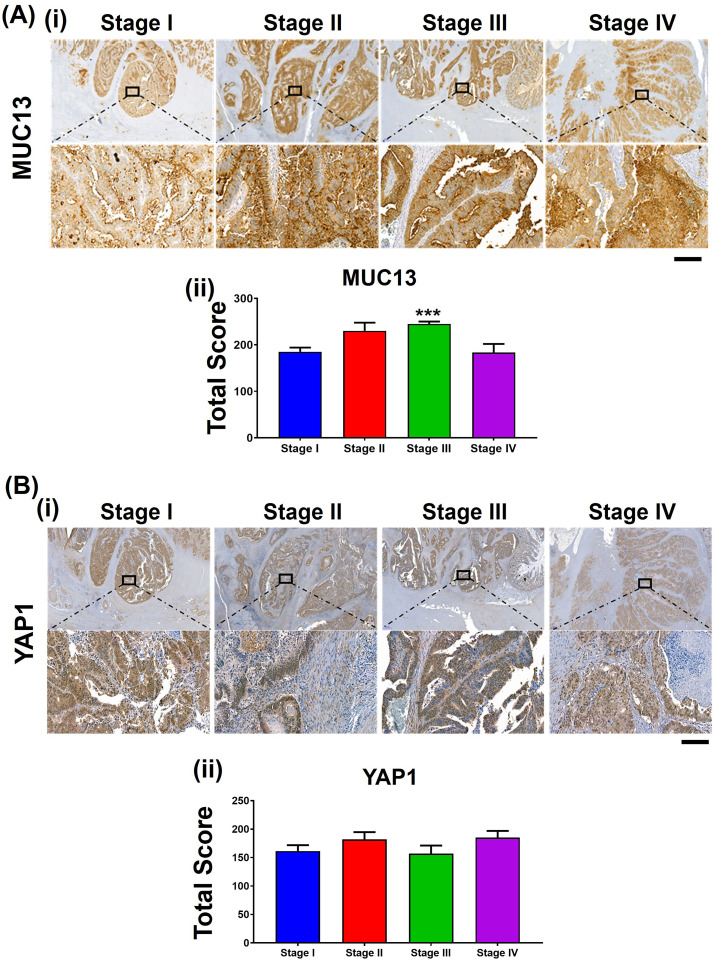
Expression of MUC13 and YAP1 in human CRC tissues. **(A, B)** (i, ii) IHC staining of MUC13 and YAP1 in different stages of human CRC tissues (scale bar, 50 μm) and (A, B) (ii) the quantitation of their total score. Unpaired *t* test. The data are shown as mean ± S.D. *P < 0.05, **P < 0.01, and ***P < 0.001 denote significant differences.

**Figure 7.**
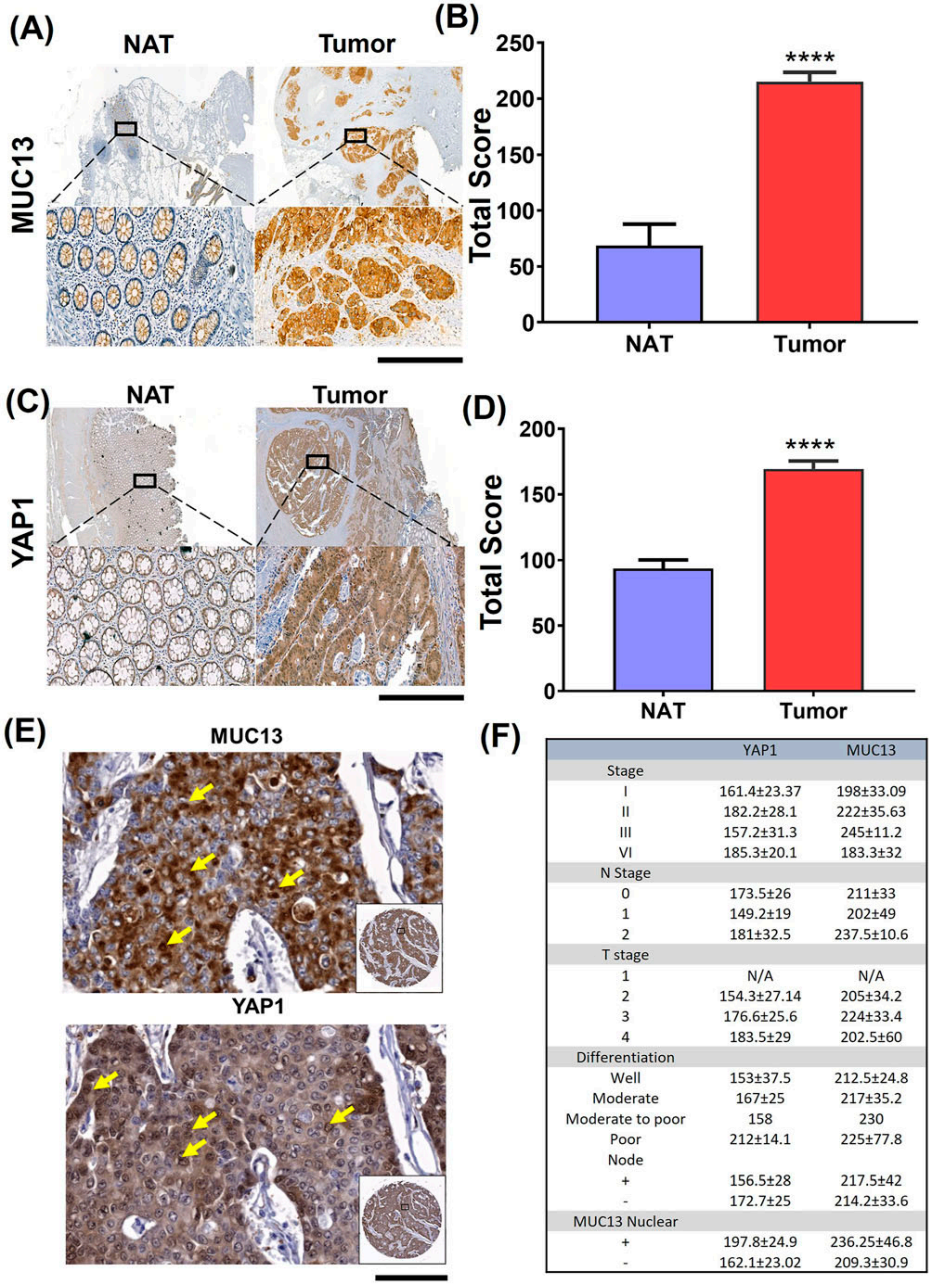
MUC13 and YAP1 expression positively correlate in human CRC tissue. **(A, B)** MUC13 IHC staining profile in NAT tissues compared with the tumor (scale bar, 50 μm) and quantitation of their respective staining scores. **(A, B, C, D)** YAP1 IHC staining profile in NAT tissues as compared with the tumor (scale bar, 50 μm) and quantitation of their respective staining score (A, B) IHC staining of MUC13 and YAP1 in different stages of human CRC tissues and (C, D) the quantitation of their total score. Unpaired *t* test. The data are shown as mean ± S.D. *P < 0.05, **P < 0.01, and ***P < 0.001 denote significant differences. **(E)** Nuclear staining of MUC13 and YAP1 in human CRC tissues of the same patients (scale bar, 20 μm). Yellow arrows indicate nuclear staining of MUC13 and YAP1 in the zoomed areas. **(F)** Clinical characteristics of human CRC tissues along with MUC13 and YAP1 staining. The average score of MUC13 and YAP1 staining in human CRC tissue samples was broken down by the stage, the N and T stage, the differentiation of the tumor, the nodal status of the tumor, and the presence of MUC13 in the nucleus.

To define the clinical significance of MUC13 and YAP1 molecular interactions, the expression of these proteins was evaluated in well-annotated human CRC tissues. Previous studies showed that the increased MUC13 expression led to nuclear localization of β-catenin, which decreased overall survival in liver cancer patients (Dai et al, 2018). In a small cohort of human CRC patient tissues, YAP1 and MUC13 showed increased expression in tumor samples (stages I, II, III, and IV) (Fig S7A and B). MUC13 and YAP1 expression was significantly higher in the tumor area compared with the adjacent normal areas (Fig 7A–D). To further define the clinical significance of this molecular interaction, the expression of MUC13 and YAP1 was further evaluated in 20 patients’ tissue across all stages. These tissues showed a significant increase in expression in the tumor when compared with the N.A.T. (Fig 7A–D). Further pathology breakdown showed that the average staining of YAP1 was higher (197) in nuclear MUC13-expressing samples compared with the nonnuclear MUC13 samples (162) (Fig 7E and F). Using a commercially available (#CO953; BioMax) CRC tissue microarray, we found that 80% of metastatic tumors (n = 10) showed nuclear expression of MUC13 and YAP1. In comparison, only 28% of the adenocarcinoma samples demonstrated nuclear expression of MUC13 and YAP1 (Table S4) (Fig 7F). These data suggest that MUC13 influences cancer metastasis and survival via nuclear translocation of YAP survival complex and its downstream oncogenic mechanisms.

Table S4. Count of number of MacroMET and MicroMET in the lungs and kidneys in tail vein-injected mice.

## Discussion

The ability of cancer cells to survive in circulation and then invade distant sites is a key aspect of metastasis. When less than 0.02% of these cells enter the bloodstream and survive, it is vital to understand the mechanism of cell survival (Fidler, 1990). MUC13 increases cell growth, migration, invasion, and colony formation of cancer cells (Chauhan et al, 2012; Gupta et al, 2014; Khan et al, 2017; Sheng et al, 2017b; Dai et al, 2018). Furthermore, with indirect activation of antiapoptotic pathways such as PI3K/AKT, MUC13 has also been shown to protect CRC cells from apoptosis (Gupta et al, 2014). Based on this, an examination of the role of MUC13 in anchorage-independent survival is of great interest. For the first time, this study reports that MUC13 plays a vital role in anchorage-independent survival of CRC cells through interaction and nuclear translocation of the YAP1-mediated survival complex, thereby inducing the expression of downstream target oncogenes.

An anchorage-independent in vitro survival model was established to mimic the fate of tumor cells in circulation and investigate molecular mechanisms operating during this condition. Two isogenic CRC cell lines (SW480; originating from a primary tumor, and SW620; originating from a metastatic lesion) were chosen for this investigation. In this anoikis model, SW480 cells exhibited 60% cell death at 36 h. In comparison, SW620 cells showed only 20% cell death under the same conditions, suggesting an inherent ability in SW620 cells to develop anchorage-independent survival (anoikis resistance). Interestingly, SW620 cells demonstrated higher MUC13 expression levels than SW480 cells at specified time points during anchorage independence. This indicates that cells with greater MUC13 expression are more impervious to loss of contact with the ECM pathway and that MUC13 might play a role in the survival of the cells. Thus, to mechanistically define the role of MUC13 in anoikis resistance, we generated MUC13 overexpressing and MUC13 knockdown stable cell lines. MUC13 overexpression in SW480 cells drastically improved the survival of these cells in the anoikis model, whereas MUC13 knockdown in SW620 cells reduced their survival. In addition to survival, MUC13 expression influenced the spheroid formation capabilities of SW480 and SW620 cells and the number of live cells in the spheroids after being introduced to anchorage-independent survival for 36 h. Furthermore, these phenotypes were correlated with the sustained expression of MUC13. These phenotypes lead to increased expression of the apoptotic relevant protein Bcl2 and decreased expression of the apoptotic end point protein caspase cleavage during the survival of cells under anchorage-independent conditions. These data confirm the vital role of MUC13 in cancer cell survival during anoikis.

Proteomics and kinome array assays on high (SW620) and low (SW480) MUC13-expressing cells were performed to elucidate the precise molecular mechanisms by which MUC13 protects cells from cell death in anchorage-independent environments. These assays suggested the differential expression of several survival and metastasis-associated genes, including the Hippo pathway effector *YAP1*, a potent transcriptional coactivator (Liu et al, 2021). The influence of MUC13 on YAP1 expression was further revealed in ectopic MUC13 expression and MUC13 knockdown cells. Based on this observation, the molecular interaction between these proteins was investigated. PLA and IP studies suggest a novel molecular interaction between MUC13 and YAP1. Tyrosine^357^ phosphorylation of YAP1 (Y^357^-YAP1) is associated with the overall survival but not the S^127^-YAP1 (Rosenbluh et al, 2012) and our data demonstrated that the ectopic MUC13 overexpression influences this phosphorylation (Y^357^-YAP1) during anoikis. MUC13 expression influenced the nuclear translocation of YAP1 and enhanced MUC13-YAP1 molecular complexation in the nucleus. Tu et al (2019) have described the role of *YAP1* as an oncogene and as a major driver of squamous subtype pancreatic ductal adenocarcinoma. In this study, they have shown that WNT5A overexpression leads to the activation of YAP1 (Rosenbluh et al, 2012). This study suggests that YAP1 activation requires additional factors, and our study demonstrates MUC13 as one of the YAP1 activators, a novel ligand for YAP1 phosphorylation, and its nuclear translocation.

YAP1 is also known to form a survival complex β-catenin, a key molecule of the Wnt signaling pathway involved in CRC (MacDonald et al, 2009; Konsavage et al, 2012; Rosenbluh et al, 2012; Herbst et al, 2014; Krausova & Korinek, 2014; Basu et al, 2016). Kinome array analysis showed the aberrant expression of several Wnt signaling pathway-associated genes, including *pGSK3-α/β* and *β-catenin*. Thus, the investigation of the influence of MUC13 on the YAP1/β-catenin survival complex was investigated. Ectopic MUC13 expression enhanced the nuclear translocation/localization of the YAP1/β-catenin survival complex. Interestingly, repression of MUC13 prevented the entry of this survival complex into the nucleus, as it was predominantly observed on the periphery of the nuclear envelope and altered the expression of their downstream survival/metastasis-associated genes. On the other hand, experimental knockdown of *YAP1* reduced cell survival, invasion, and migration. This finding clearly suggests a critical role for MUC13-YAP1 in cancer cell survival and metastasis processes. A recent study indicates that MUC13 plays a role in β-catenin-mediated Wnt signaling in liver cancer (Dai et al, 2018). Our study defined three main points: (1) MUC13 forms a molecular complex with YAP1; (2) MUC13 promotes nuclear shuttling of YAP1/β-catenin survival complex and YAP1, and (3) MUC13 influences the expression of survival/metastasis-associated genes in cancer cells during the anchorage-independent survival period, providing anoikis resistance.

Cells escaping from anoikis, their extravasation, settling, and colonization are the next necessary steps for successful metastasis at distant organ sites (Paoli et al, 2013). Thus, investigating if MUC13, besides providing anoikis resistance to cancer cells, can facilitate the next steps of metastasis is vital. In vivo, metastasis studies in the tail vein injection mouse model showed that the ectopic MUC13 expression provides a metastatic advantage to the SW480−MUC13 cells, as evident from visible metastatic lesions in the kidneys, lungs, and liver. However, no micro or macrometastatic lesions were observed in SW480+vector control cells. MUC13, YAP1, and β-catenin were highly expressed in metastatic lung and kidney lesions. The nuclear expression of YAP1, MUC13, and β-catenin was also evident in the metastatic lesions. However, it was more prominent in the kidney lesions compared with the lungs. This probably explains why the kidneys showed more pronounced metastasis than the lungs and liver. Our IHC data suggest nuclear expression of MUC13 in metastatic lesions, despite being a transmembrane protein. Nuclear translocation of transmembrane proteins, including mucins (MUC1), has been demonstrated in several studies (Kufe, 2013). A recent study has also suggested that MUC13 expression promotes cellular growth in hepatocellular carcinoma via influencing Wnt signaling and its interaction with beta-catenin (Dai et al, 2018). Looking into the evidence that *YAP1* gene is a context-dependent oncodriver (Tu et al, 2019), there is a huge possibility that MUC13 alone or along with YAP1 can translocate into the nucleus during the anoikis resistance and metastasis process. These data suggest that MUC13 is critical in facilitating YAP1-mediated oncogenic and metastatic signaling pathways in cancer cells. Higher expression of MUC13 and YAP1 was observed at different tumor stages compared with adjacent normal samples, highlighting the clinical significance of this novel molecular interaction. Interestingly, tumor samples that showed nuclear MUC13 expression also demonstrated higher YAP1 expression. This strengthens the argument that MUC13 is critical in driving cancer aggressiveness and metastasis through the YAP1-dependent oncogenic pathway. Thus, the biochemical intervention of this new molecular interaction can uniquely treat cancer metastasis.

In conclusion, using an anoikis model, an in vivo metastatic model, and CRC patient samples, this study identified a novel molecular mechanism that provides anoikis resistance to detached tumor cells and facilitates their metastasis at distant organ sites. Furthermore, this study reveals that MUC13 plays a critical role in these metastasis-associated processes via interaction with YAP1 and nuclear translocation of the YAP1-mediated survival complex. Therefore, a biochemical intervention that interferes with MUC13–YAP1 complex formation can help develop new therapeutics for the treatment of metastatic cancer.

## Materials and Methods

### Plasmids, cell culture, and lentiviral transduction

Lentiviral Gen III packaging plasmids pMDLg/pRRE encoding gag and pol (#12251), pRSV-Rev encoding Rev (#12253), and pMD2.G encoding vesicular stomatitis virus G protein (VSV-G envelope) (#12259) were procured from Addgene. MUC13 shRNA plasmids in pLKO.1 backbone were procured from Sigma-Aldrich (TRCN0000429044; Clone ID: NM_033049.2-1949s21c1, sequences in Table S5). Lentiviral MUC13 expression plasmids (1,539-bp human NM_033049.4) were procured from Abmgood (LV230821) along with an empty vector, and plasmids were isolated using the Promega Midi kit (Cat #A7640; Promega) per manufacturer instructions. Plasmids were transfected into log-phase cells. A Gen III Lentiviral system was used to produce packaged virus particles using HEK293T cells. Concentrated supernatant containing packaged lentiviral particles transduced SW620 cells for MUC13 knockdown and SW480 for MUC13 overexpression. These cells were selected with 1.5 μg/ml puromycin SW480+Vec, SW480+MUC13, SW620+shVec, and SW620+shMUC13 stable cell lines, maintained in 0.2 μg/ml of puromycin and characterized.

Table S5. List of shMUC13 sequences used in this study.

### Anoikis induction

Anoikis was induced by plating cells on poly (2-hydroxyethyl methacrylate) (poly-HEMA; Cat #P3932; Sigma-Aldrich)-coated tissue culture plates. 4 ml of poly-HEMA solution (20 mg/ml in 95% ethanol) was used per 10-cm tissue culture plate and dried overnight in a tissue culture hood. For anoikis induction, cells were plated in appropriate media at a density of 5 × 10^6^ per plate and collected at 24, 36, and 48 h time points for different assays.

### Real-time quantitative PCR assays

Total RNA samples extracted using Trizol were processed for real-time PCR (Light Cycler 480; Roche) using specific primers for MUC13, Bcl2, c-Myc, Aaxin 2, and Cyclin D1 and SYBR Green Master Mix (cat #KM4106; Kapabiosytems). β-Actin was used as an internal loading control, and expression levels were normalized to that of β-actin and calculated using the delta–delta Ct method. The assays were performed in quadruplicate, and the experiment was repeated three times. A PCR primer list is provided in Table S6.

Table S6. Primer sequences used in this study.

### Immunoblotting assay

Anoikis-induced cells collected at different time points were harvested, washed in 1xPBS, and lysed in RIPA buffer (50 mmol/liter Tris, pH 7.5, 150 mmol/liter NaCl, 1% NP-40, 0.5% Na-deoxy Cholate, 0.1% S.D.S.) containing a cocktail of protease and phosphatase inhibitors. Whole-cell extracts, after quantification, were fractionated on 4–12% gradient SDS–PAGE and processed for immunoblotting using anti-MUC13 (Cat#NBP2-25466; Novus), anti-Bcl2 (Cat#2872; Cell signaling), anti-cleaved caspase 3 (Cat#9662; Cell Signaling), anti-β-Actin (Cat #A5441; Sigma-Aldrich), anti-YAP1 (Cat #52771; Abcam), and anti-β-catenin (Cat#610154; BD Biosciences). After washing in TBST, antibody–antigen complexes were detected using horseradish peroxidase-conjugated goat anti-rabbit or mouse (Cat #W4011, W4021; Promega) secondary antibody with an ECL chemiluminescence (cat #WBKLS0500; Millipore). Signals were developed on an X-ray film or iBright Cl1500 (cat #A44240; Invitrogen) and quantitated using Image J software (https://imagej.nih.gov/ij/).

### Cell cycle assays

Cells (1 × 10^6^) were grown on poly-HEMA-coated six-well tissue culture plates for anoikis induction. After 24, 36, and 48 h, the cells were pelleted, washed in PBS, and fixed in cold 70% ethanol overnight at −20°C. Fixed cells were stained with Telford Reagent (Cat #P-4170; Sigma-Aldrich) at 4°C for 6 h. The Bio-Rad ZE5 Analyzer was used for flow cytometry analysis. Cells displaying hypo-diploid DNA were regarded as apoptotic (SubG0/G1). Data from 10,000 cells were collected and analyzed using ModFit LT version 2.0 for cell cycle analysis.

### IHC

The sectioned FFPE human CRC and organs containing metastatic nodules (FFPE) from the tail vein experiment were probed with anti-YAP1 (Cat #52771; Abcam), anti-β-catenin (Cat #610154; BD Biosciences), and anti-MUC13 (inhouse C14 mAb) antibodies as described earlier using a polymer-based MACH4 IHC kit (Biocare Medical). Briefly, tissues were heated, deparaffinized, and rehydrated with graded ethanol treated with peroxidase solution. Antigen retrieval followed by primary/secondary antibody treatment, intermittent washings, and tissue sections were finally developed using a 3,3′-diaminobenzidine reagent (DAB) solution for 2 min.

No primary antibody was considered a control for the specificity of IHC staining. After counterstaining with hematoxylin and mounting, the slides were scanned through a digital 3D Histech (Aperio) scanner platform. Results were analyzed using case viewer software (3D Histech) with the help of a trained pathologist.

### Spheroid assay

Anoikis-induced cells harvested after 36 h, pelleted, and suspended in cultured media were co-cultured with 3T3 mouse fibroblast at a ratio of 2:1 in an ultra-low attachment 96-well plate. After 168 h, the spheroids were stained using the LIVE/DEAD Cell Imaging kit (Cat #R37601; Invitrogen) for 15–20 min, and phase contract/GFP/Texas Red images were acquired using the Cytation 5 Cell Imaging Multi-Mode Reader (Bio Tek). Image J software was used to calculate the fluorescence.

### Immunofluorescence and PLA

Anoikis-induced cells were pelleted, washed, and fixed in 4% paraformaldehyde. Fixation was quenched using 100 mM glycine pellets, which were processed for cryo-sectioning using OCT media and a cryostat. ∼8–10-μm thick sections on slides were used for immunocytochemistry and PLA studies. Briefly, sections were permeabilized with 2% Triton X-100, blocked in 10% donkey serum, and then incubated overnight in primary antibodies (anti-β-catenin mAb [Cat #610154; BD Biosciences], anti-YAP1 mAb [Cat #52771; Abcam], and anti- MUC13 [Cat#NBP2-25466; Novus]). After washing, samples were incubated with Cy3 anti-rabbit and Alexa 488 anti-mouse secondary antibodies. Slides were mounted with Vector Shield with DAPI (Cat#H-2000; VECTASHIELD), and images were captured at 400x magnification using a confocal microscope (Nikon Corporation). Images were analyzed using free Zen software provided by Zeiss and Image J software.

#### PLA

Anoikis-induced cells were cryofixed and used in situ PLA to detect protein–protein interactions (Duolink). Cryo-fixed control and experimental cells on the slides were stained with MUC13/β-catenin; YAP1/β-catenin; YAP1/MUC13; and β-catenin/MUC13 antibody set. For specificity, a negative control (no primary antibody) was included. The amplification was carried out using the DuolinkTM in situ complementary oligonucleotide probes MINUS and PLUS 5x, secondary antibody conjugated with a PLA oligonucleotide, anti-rabbit PLUS, anti-mouse MINUS, anti-rabbit MINUS, and anti-mouse PLUS (catalog number: DUO82029, DUO 92004, 92001, 92005; Sigma-Aldrich). All the antibodies used for PLA were verified by utilizing the PLUS and MINUS probes of the same species as the primary antibodies. The verified antibodies were taken as pairs (mouse and rabbit) to detect protein–protein interaction. Images were acquired at 40x magnification using a confocal microscope (Nikon Corporation) and processed with MetaMorph software.

### Human kinase array

Anoikis-induced control and experimental cell lysates were prepared according to the Human Phospho-Kinase Array kit (Cat # ARY003B; R&D Systems) manual. Detection antibodies (cocktails A and B) and streptavidin-HRP second antibody were used to probe the membrane, developed with Chemi Reagent Mix, and imaged to film using the iBright Cl1500 imaging station (cat #A44240; Invitrogen).

### Proteomic analysis

Control SW480 and experimental SW620 cells were grown on low attachment plates for 36 h at 70% confluency to induce anchorage-independent survival pathways. A QC experiment check was performed by analyzing the differential expression of MUC13 and other marker protein expression using Western blot (data not shown) before submitting them for the proteomic analysis at the UTHSC Proteomics Core. The 36-h-grown cells in two groups (three samples per group) were analyzed using iTRAQ (isotope-Coded Affinity Tags) with an LTQ-Orbitrap mass spectrometer. Briefly, cells in two groups were lysed using Cell Lysis buffer from Pierce Mass Spec Sample Prep Kit for Cultured Cells (P/N 84840) in a total volume of 20 μl, protease digested, and the resulting peptide products from each sample were labeled with a different isobaric tag. MS and Bioinformatics analyzed the labeled samples. The UTHSC Bioinformatics Core analyzed raw mass spec data using the iPathway Guide (Advaita Bioinformatics). A 1.5-fold difference was set as the analysis’s cutoff for significant fold change.

### Invasion and migration analysis

Transwell migration and Matrigel invasion analyses of YAP1 knockdown in MUC13 overexpression cells (SW480−MUC13+shYAP1) were performed, as mentioned earlier (Ganju et al, 2018).

### Animal studies

All animal studies were approved by the University of Tennessee Institutional Animal Care and Use Committee (IACUC) and performed according to the Association of Assessment and Accreditation of Laboratory Care standards. Anoikis-induced control and experimental cell lines were pelleted, washed, and passed through a 70-μm strainer. These cells (1 × 10^6^ cells per 100 μl; SW480+Vec and SW480+MUC13) were injected into the tail vein of NSG (NOD- Cg-Prkdc<scid> Il2rg<tm1Wjl>/SzJ) mice. Mice were monitored weekly; after 4.5 wk, mice were euthanized, and organs were analyzed for metastatic nodules. Excised organs/tumors were evaluated using IHC for different markers.

### IHC analyses with human tissues

Human CRC tissue (FFPE) sections were procured from the Department of Pathology, the University of Tennessee Health Science Center (Memphis, TN, USA), per UTHSC IRB guidelines. In this study, archival FFPE tissues were used, and the tissue samples were coded to protect patient privacy. The research was carried out in compliance with the Declaration of Helsinki, and the “Human Subject Exempt procedure” was approved by the I.R.B. Committee of the University of Tennessee at Memphis, Memphis, Tennessee, USA; project identification number 13-02690-XM, authorized on 28 August, 2013.

### Statistical analysis

GraphPad Prism 7 (GraphPad Software; GraphPad) was used for statistical analyses of all experimental procedures. Assays comprising two groups with equal or unequal variance, unpaired two-tailed *t* tests, or *t* tests with Welch’s correction were performed, respectively. Two-way ANOVA statistical tests were performed in instances with three or more groups, followed by Sidak’s multiple comparison tests for pairwise analysis. In all cases, a *P*-value of <0.05 was considered statistically significant.

## Supplementary Material

Reviewer comments
